# Occurrence of haemolytic *Mannheimia *spp. in apparently healthy sheep in Norway

**DOI:** 10.1186/1751-0147-48-19

**Published:** 2006-10-31

**Authors:** Louise L Poulsen, Turið M Reinert, Rikke L Sand, Magne Bisgaard, Henrik Christensen, John E Olsen, Snorre Stuen, Anders M Bojesen

**Affiliations:** 1 Department of Veterinary Pathobiology, The Royal Veterinary and Agricultural University, 4 Stigbøljen, DK-1870 Frederiksberg C, Copenhagen, Denmark; 2 Norwegian School of Veterinary Science, Department of Production Animal Clinical Sciences, Sandnes, Norway

## Abstract

**Background:**

The occurrence of *Mannheimia *species in healthy sheep has only been investigated to a very limited extend since the genus and its five named species were established. The aim of the present study was to evaluate the occurrence of haemolytic *Mannheimia *species in apparently healthy sheep originating from four sheep flocks in South-Western Norway.

**Methods:**

Typical *β*-haemolytic *Pasteurellaceae *were isolated from nasal swabs and subsequently subjected to bacteriological examination. A total of 57 *Mannheimia *isolates were obtained in pure culture. All isolates were genotyped by amplified fragment length polymorphisms (AFLP) analysis and compared to six reference strains. The 16S rRNA gene sequences of two isolates were also determined.

**Results:**

*β*-haemolytic *Mannheimia *species were isolated from 24% to 64% of the sheep in the four flocks. A total of 26 haemolytic *M. ruminalis*-like strains were isolated among which, a considerable genetic diversity was found. Eighteen *M. glucosida *isolates were obtained from three flocks, whereas *M. haemolytica *was only isolated from two flocks, 16 of them being from only one of the flocks.

**Conclusion:**

We demonstrate that a relatively high number of apparently healthy sheep in Norway seem to carry the potentially pathogenic *M. haemolytica *and *M. glucosida *in the upper respiratory tract. An unexpectedly high number of haemolytic *M. ruminalis*-like organisms were also obtained in all four flocks. The usually non-haemolytic *M. ruminalis *are typically isolated from healthy ruminants. The significance of *β*-haemolytic *M. ruminalis*-like organisms is unknown and should be investigated in a future study.

## Background

*Mannheimia *was proposed for the trehalose-negative *[Pasteurella] haemolytica *complex including at least seven species, five of which were named [1]. *[P.] haemolytica *was reclassified as *M. haemolytica *including the former *[P.] haemolytica *biogroup 1 and serovars 1, 2, 5, 6, 7, 8, 9, 12, 13, 14 and 16 while *M. glucosida *was proposed to include *[P.] haemolytica *serovar 11, biogroups 3A-H and the *β*-glucosidase and meso-inositol positive strains of biogroup 9. *M. varigena *includes the former *[P.] haemolytica *biogroup 6 and Bisgaard taxon 15 and taxon 36. The former *[P.] granulomatis *and Bisgaard taxon 20 and *[P.] haemolytica *biogroup 3J were combined in *M. granulomatis*. Finally, *M. ruminalis *was proposed to include the former Bisgaard taxon 18 and *[P.] haemolytica *biogroup 8D [1]. A novel *Mannheimia *species was subsequently reported from Australian feedlot cattle [2]. The specificity of serotyping as a diagnostic tool was subsequently investigated by Angen *et al*. [3,4] who demonstrated that serotyping does not represent a reliable method for identification. The same authors emphasized that extended phenotypic and genetic characterization is necessary for proper identification of these organisms making it difficult to decide which of the present taxa are dealt with in previous studies, based only upon phenotyping and serotyping [5-11].

Most species of *Mannheimia *are known as opportunistic pathogens [12] and are frequently isolated from asymptomatic carriers [6,9,13]. In sheep, disease caused by *Mannheimia *species has mainly included pneumonia and septicaemia [13], although isolates have been reported from cases of mastitis and the myocardium and the brain of healthy animals [3].

Due to the above mentioned changes in taxonomy and the impact it may have on the understanding of the aetiology, pathogenesis and epidemiology of these organisms, the present investigation aims at determining the occurrence of haemolytic *Mannheimia *species in healthy sheep herds from South-Western Norway. The results demonstrated that a relatively high number of apparently healthy sheep in Norway seems to carry the potentially pathogenic *M. haemolytica *and *M. glucosida *in the upper respiratory tract. In addition we found an unexpectedly high number of haemolytic *M. ruminalis*-like organisms in all four flocks.

## Materials and methods

### Herds and bacterial sampling

A total of 139 adult sheep originating from four herds in Ims, Stave, Kjosavik, and Sandnes, Norway were included in the study which took place during the spring of 2005. Epidemiological relations were not demonstrated among the randomly selected herds. The herd in Ims consisted of 33 adult Texel sheep. Within this herd, a single sheep suffered from pasteurellosis in 2004. A new ram was introduced the same year, but otherwise the flock had had no contact with other sheep. The flock in Stave included 38 adult Norwegian white sheep. Four or five cases of pasteurellosis were observed during 2004. A new ram was introduced to the flock the same year and part of the flock had been grazing with other herds during the summer. The Kjosavik flock consisted of 40 adult Norwegian white sheep and crossbreeds. A single animal suffered from pasteurellosis in 2004. No new animals were introduced to the flock during 2004, but the flock had been grazing in the mountains with other herds during the summer. Finally, the flock in Sandnes included 200 adult sheep of various mixed breeds of Norwegian White Sheep. From this herd, 11 adult sheep and 17 lambs were included in the survey. Though, the herd was grassing with other herds during the summer, pasteurellosis had not been diagnosed in this flock for many years. The tentative diagnosis, pasteurellosis was based on the lesions described by Gilmour and Gilmour [13].

Bilateral nasal swabs were taken using sterile cotton swabs from each animal. The swabs were plated onto 5% ovine blood agar plates (Oxoid) and incubated in 5.8% CO_2 _for 18–20 hours at 37°C. After incubation, the plates were examined for *Pasteurellaceae*-like colonies that were greyish or yellowish, and showing a narrow zone of *β*-haemolysis. A single haemolytic colony was subcultured and tested by Gram-staining, catalase, and oxidase. All strains which proved catalase positive, cytochrome-oxidase positive, Gram-negative and having the typical colony morphology of *Pasteurellaceae *were stored at -80°C in porous beads (Microbank™, pro-lab diagnostics) and subsequently taken to The Department of Veterinary Pathobiology, The Royal Veterinary and Agricultural University, Copenhagen for further characterization.

### Bacterial phenotyping

A total of 57 pure cultures were characterised phenotypically. Isolates were designated I for Ims, S for Stave, K for Kjosavik and only the number from Sandnes (except for C1, also from Sandnes). Phenotypical characters included, colony morphology on blood agar (Tryptose blood agar base [Difco] containing 5% citrated bovine blood), cell morphology observed by phase-contrast microscopy, Gram-staining, 3% KOH test, catalase (3% H_2_O_2_) and oxidase (Fluka). Motility was examined by phase-contrast at 37°C, and by inoculation in VL-semisolid medium (Heart Infusion Broth (Difco) added 0. 25% agar) following incubation at 22°C for 24 hours. Catabolism of glucose was examined in Hugh and Leifson's medium (Merck). Urease was tested in Christensen's urea medium (Merck). Indole production was examined by adding Kovac's reagent to a 48 hours old Heart Infusion Broth culture. Production of acid from: L(+)arabinose, D(+)mannose, and trehalose was examined as reported previously [14] while production of α-fucosidase (ONPF) and β-glucosidase (NPG) were determined with Rosco diagnostic tablets according to the manufactures instructions. CAMP-test was performed according to Christie *et al*. [15].

### Bacterial genotyping by AFLP and 16S rRNA sequencing

Isolates classified with species of *Mannheimia *were subsequently characterized by AFLP and compared with the following reference strains: *M. haemolytica *NCTC 9380^T^, *M. glucosida *P925^T ^and *M. ruminalis *HPA92^T^. Furthermore, six strains previously identified as *M. ruminalis *(HPA90, HPA93, HPA98, HPA109) and *M. glucosida *(P730, P733) were included [16].

DNA was extracted using a DNA purification kit (Prestospin D Bug, Molzym) according to the manufacturer's instruction. AFLP typing was carried out as reported previously [17]. Briefly, the non-selective *Bgl*II primer (FAM-5' GAGTACACTGTCGATCT 3') and the non-selective *Bsp*DI primer (5' GTGTACTCTAGTCCGAT 3') were used to amplify the fragments subsequent to restriction digestion and ligation to their corresponding adaptors. Amplification products were detected on an ABI 377 automated sequencer (PE Biosystems). Each lane included an internal-lane size standard labelled with ROX dye (Applied Biosystems) and GeneScan 3.1 fragment analysis software (Applied Biosystems) was used for fragment size determination and pattern analysis. AFLP profiles comprising fragments in the size range 50–500 bp were considered for numerical analysis with the program GelCompar II (Applied Maths, Kortrijk, Belgium). Normalised AFLP fingerprints were compared using the Dice similarity coefficient and clustering analysis was performed by the UPGMA.

**Figure 1:**
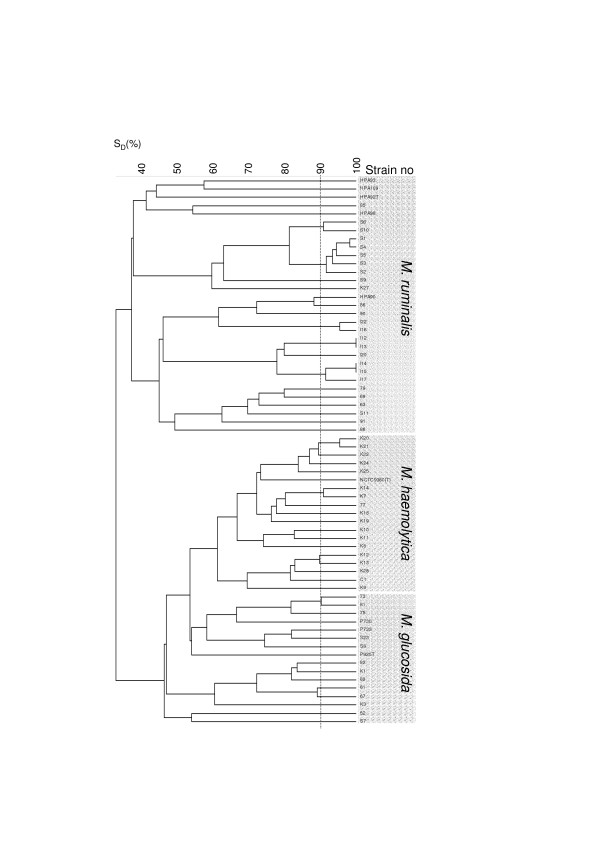
**Genetic diversity among the obtained *Mannheimia *isolates**. Dendrogram showing AFLP similarities (Dice coefficient) between the *Mannheimia *isolates obtained from healthy sheep and the type strains *M. haemolytica *(NCTC9380^T^), *M. glucosida *(P925^T^) and *M. ruminalis *(HPA92^T^). Six strains previously identified as *M. ruminalis *(HPA90, HPA93, HPA98, HPA109) and *M. glucosida *(P730, P733) were also included. Isolates obtained in the present study were designated I from Ims, S from Stave, K from Kjosavik and only the number from Sandnes (except C1 also from Sandnes). The vertical dotted line at 90% similarity denotes the cut-off value for a clone.

16S rRNA gene sequencing included two strains, S9 and 85, representing two of the three main clusters in the *M. ruminalis *cluster in Fig. 1. PCR amplification was performed according to the standard conditions described by Vogel *et al*. [18]. Oligonucleotides for both PCR amplification and 16S rRNA sequencing were synthesized according to sequences and positions given in Dewhirst *et al*. [19] and Paster and Dewhirst [20]. DNA sequencing was performed on the ABI 377 (Applied Biosystems) sequencer with unlabelled primers and the BigDye kit according to protocols described with the Chemistry Guide for automated DNA sequencing (Applied Biosystems). Searches for DNA sequences at NCBI were performed by BLAST [21].

## Results

Based on the phenotypic characters outlined for each *Mannheima *species in Table 1, the species distribution of isolates obtained from the present investigation is summarized in Table 2. There were found no discrepancies between the phenotypic character demonstrated for present isolates and the ones outlined for the different *Mannheimia *species by Angen *et al*. [1]. However, given the considerable genetic diversity among the isolates classifying with *M. ruminalis *will be referred too, as *M. ruminalis*-like organisms. Interestingly, all isolates classified with *M. ruminalis *were *β*-haemolytic on ovine blood, although this species generally is regarded as non-haemolytic. The *M. ruminalis*-like organisms were the most prevalent *Mannheimia *species present in all four flocks.

**Table 1:** Phenotypic characters used for identification and separation of species investigated.

	*M. haemolytica*	*M. glucosida*	*M. ruminalis*
Gram-staining	-	-	-
Catalase	+	+	+
Oxidase	+	+	d
Motility	-	-	-
Haemolysis on ovine blood	+	+	d
Haemolysis on bovine blood	+	+	-
H&L, glucose	F	F	F
Urease	-	-	-
Indole	-	-	-
D(+)mannose	-	-	-
Trehalose	-	-	-
L(+)arabinose	-	d	-
α-fucosidase	+	+	-
β-glucosidase	-	+	-
CAMP reaction	+/weak	+	-

+: ≥ 90% strains are positive, -: < 10% strains are positive, d: <90 and ≥10 positive, (+): Late positive (3–14 days), F: fermentative [1].

**Table 2:** Species identified within the four herds investigated.

Farm	Flock size	*M. haemolytica*	*M. ruminalis*	*M. glucosida*	No of sheep affected with pasteurellosis in previous years
Ims	33	-	8	-	1
Stave	38	-	9	3	4/5
Kjosavik	40	16	1	2	1
Sandnes	200 ^*^	2	8	8	0

^* ^(28 sampled)

AFLP typing and cluster analysis revealed two major clusters diverging at a 38% similarity level, one containing isolates classified with *M. ruminalis *and another containing isolates classified as *M. haemolytica *and *M. glucosida*. All 26 isolates of *M. ruminalis*-like isolates clustered with the type strain and the four additional strains of *M. ruminalis *included. A considerable genetic diversity was observed within the *M. ruminalis *cluster. For this reason two isolates (S9 and 85) each representing a subcluster containing the *M. ruminalis *type strain (HPA92^T^) and a subcluster including no reference strain, respectively, were characterized by 16S rRNA gene sequencing. The results showed 99.6% and 99.4% similarity between the type strain, *M. ruminalis *(HPA92^T^), and the isolates S9 and 85, respectively. A 99.7% 16S rRNA sequence similarity was observed between the isolates S9 and 85. In addition, these isolates showed 96.5% to 98.1% 16S rRNA sequence similarity with the type strains of *M. haemolytica*, *M. glucosida *and *M. granulomatis*, the highest similarity observed between S9 and the *M. glucosida *type strain.

The genetic diversity of the *M. ruminalis*-like isolates obtained from the Sandnes flock was considerable while isolates from the three other farms were generally more closely related. Six out of eight isolates of *M. ruminalis*-like isolates from the flock in Ims were highly related (Fig. 1). Only around 38% similarity was observed between the type strain and the isolates obtained in the present study.

Sixteen out of 40 sheep from the flock in Kjosavik sampled positive for *M. haemolytica *while only two (77 and C1) sheep out of 28 examined from the flock in Sandnes sampled positive. All 18 *M. haemolytica *isolates formed a separate AFLP cluster with the type strain of *M. haemolytica *(NCTC9380^T^). A total of 13 isolates of *M. glucosida *were obtained from the flocks in Stave, Kjosavik, and Sandnes. These isolates also formed an AFLP cluster grouping with the type strain (P925^T^) and the included reference strains (P730 and P733).

## Discussion

Previous investigations on the prevalence of [*P*.] *haemolytica *have shown a considerable variation. A range between 8.9% and 96.2% of healthy sheep that carry these organisms in the nasal cavity have been reported [6,7,22]. The variation is likely to be caused by several factors including different isolation techniques, misidentification, and seasonal variation. Swabbing of the tonsils and nasal cavity of slaughtered sheep showed that *[P.] haemolytica *could be isolated from 95% of the tonsils and 64% of the nasopharyngeal swabs [9]. Furthermore, it has been found that the prevalence of *[P.] haemolytica *in temperate climates varies seasonally with a higher prevalence in spring and early summer [13].

In the present survey, the isolation rate of *Mannheimia *spp. varied from 24% to 64% in the four herds investigated. However, only a single haemolytic colony on sheep blood agar was subcultured and characterized from each sample. In addition, the nasal cavity was sampled instead of the tonsils which might explain the complete lack of isolation of [*P*.] *trehalosi*. In this survey, the sheep were swabbed in May, where the prevalence of *Mannheimia *spp. was expected to be at its highest. With the exception of the flock in Ims, at least two different species of *Mannheimia *were isolated in each flock. In addition, six out of eight isolates of *M. ruminalis *from the flock in Ims were highly related genetically, probably reflecting the fact that this flock has been kept isolated over time. This flock also showed the overall lowest isolation rate of *Mannheimia *spp.

According to Gilmour and Gilmour [13], [*P*.] *haemolytica *(*M. haemolytica*) is normally associated with pneumonia in cattle and sheep, septicaemia in lambs and mastitis in ewes. These observations have subsequently been supported by Angen *et al*. [1,4]. However, the present investigation clearly demonstrated that these organisms also can be obtained from the upper respiratory tract of apparently healthy sheep.

*M. glucosida *seems to be associated with the upper respiratory tract and rumen of sheep, but has also been reported from various lesions [1,4]. *M. ruminalis *has, so far only been reported from the rumen of sheep and cattle [1,3] and mainly in a non-haemolytic form. However, only relatively few strains are available and considering the results of the present study, it may be questioned whether the non-haemolytic phenotype is general to *M. ruminalis *or if this merely applies to a sub-population of this species. We feel confident about the classification of the *M. ruminalis*-like isolates from this investigation as they showed very high 16S rRNA gene similarity to the *M. ruminalis *type strain (HPA92^T^) and also clustered with the *M. ruminalis *strains HPA98 ([*P*.] *haemolytica *biogroup 8), HPA90 (Bisgaard taxon 18 biovar 2 (*arab*^+^)) and HPA93 and HPA109 (Bisgaard taxon 18 biovar 3 (*sorb*^+^)), as demonstrated by AFLP. Except for the haemolytic phenotype of the *M. ruminalis*-like strains there were no discrepancies to the phenotypic results outlined in Table 1. It has been suggested that the non-haemolytic *M. ruminalis *have lost virulence factors associated with their pathogenic and phylogenetic ancestors [23]. It could therefore be speculated if the *β*-haemolytic *M. ruminalis-*like organisms isolated in the current study represent an intermediate form. Alternatively, there may be different subpopulations, which have adapted to different niches i.e. in the nasal cavity and rumen. Although our study included representatives from both the nasal cavity and the rumen we were not able to demonstrate genetically distinct sub-populations based on the site of isolation. Loss of virulence factors may reflect that these organisms generally have adapted to a broader ecological niche, which is supported by the high genetic diversity demonstrated by AFLP. Consequently, future studies addressing the *β*-haemolytic phenotype and its genetic background in *M. ruminalis *will be highly relevant.

Savelkoul *et al*. [24] found that *Klebsiella *strains showing a 90–100% fingerprint similarity could be considered as belonging to the same clone. In other studies including *Acinetobacter *[25] and *Gallibacterium anatis *[26] the same similarity values have been used to define a clone. Provided that the same value can be used to define clones within species of *Mannheimia*, it indicated that all flocks investigated have been exposed to several introductions of *Mannheimia *spp. over time or that several clonal lineages have evolved within each flock.

## Conclusion

The present study demonstrates that *M. haemolytica, M. glucosida *and *M. ruminalis*-like organisms are commonly found in the upper respiratory tract of healthy sheep. Interestingly, the occurrence of haemolytic *M. ruminalis*-like isolates was unexpectedly high. This organism has usually been isolated in a non-haemolytic form. The importance of this finding will have to be investigated in future studies, in order to conclude on the biological significance of the different haemolytic/non-haemolytic phenotypes.

## Abbreviations

AFLP: Amplified Fragment Length Polymorphisms

UPGMA: Unweighted Pair Group Method with Arithmetic Averages

## Competing interests

The author(s) declare that they have no competing interests.

## Authors' contributions

LLP, RLS and TRM took part in all aspects of the investigation including planning, sampling, phenotypic and genotypic characterization and drafting of the manuscript. SS participated in the planning, sampling and initial characterization of the bacterial isolates. MB participated in the planning, phenotypical characterization and drafting of the manuscript. HC did the 16S rRNA sequence alignments. JEO participated in the planning and drafting of the investigation. AMB participated in the genotypical characterization, drafting and revision of the manuscript. All authors read and approved the final manuscript.

## References

[B1] Angen Ø, Mutter R, Caugant DA, Olsen JE, Bisgaard M (1999). Taxonomic relationships of the [*Pasteurella*] *haemolytica *complex as evaluated by DNA-DNA hybridizations and 16S rRNA sequencing with proposal of *Mannheimia haemolytica *gen. nov., comb. nov., *Mannheimia granulomatis *comb. nov., *Mannheimia glucosida *sp. nov., *Mannheimia ruminalis *sp. nov. and *Mannheimia varigena *sp. nov. Int J Syst Bacteriol.

[B2] Blackall P, Angen Ø, Fegan N, Blackall L, Mutters R, Bisgaard M (2001). Characterisation of a novel *Mannheimia *spp. from Australian feedlot cattle. Aust Vet J.

[B3] Angen Ø, Quirie M, Donachie W, Bisgaard M (1999). Investigations on the species specificity of *Mannheimia *(*Pasteurella*) *haemolytica *serotypning. Vet Microbiol.

[B4] Angen Ø, Ahrens P, Bisgaard M (2002). Phenotypic and genotypic characterization of *Mannheimia *(*Pasteurella*) *haemolytica*-like strains isolated from diseased animals in Denmark. Vet Microbiol.

[B5] Al Sultan II, Aitken ID (1985). The tonsillar carriage of *Pasteurella haemolytica *in lambs. J Comp Path.

[B6] Al-Tarazi YHM, Dagnall GJR (1997). Nasal carriage of *Pasteurella haemolytica *serotypes by sheep and goats in Jordan. Trop Animal Health Prod.

[B7] Biberstein EL, Shreeve BJ, Thompson DA (1970). Variation in carrier rates of *Pasteurella haemolytica *in sheep flocks I. Normal flocks. J Comp Path.

[B8] Fraser J, Gilmour NJL, Laird S, Donachie W (1982). Prevalence of *Pasteurella haemolytica *serotypes isolated from ovine pasteurellosis in Britain. Vet Rec.

[B9] Gilmour NJL, Thompson DA, Fraser J (1974). The recovery of *Pasteurella haemolytica *from the tonsils of adult sheep. Res Vet Sci.

[B10] Shreeve BJ, Thompson DA (1970). Studies on the carriage of *Pasteurella haemolytica *in lambs. J Comp Path.

[B11] Shreeve BJ, Biberstein EL, Thompson DA (1972). Variation in carrier rates of *Pasteurella haemolytica *in sheep. II. Diseased flocks. J Comp Path.

[B12] Bisgaard M (1993). Ecology and Significance of *Pasteurellaceae *in Animals. Zentralbl Bakteriol.

[B13] Gilmour NJL, Gilmour JS, Adlam C, Rutter JM (1989). Pasteurellosis of sheep. Pasteurella and Pasteurellosis.

[B14] Bisgaard M (1975). Characterization of atypical *Actinobacillus lignieresii *isolated from ducks with salpingitis and peritonitis. Nord Vet Med.

[B15] Christie R, Atkins NE, Munch-Petersen E (1944). A note on a lytic phenomenon shown by group B streptococci. Aust J Exp Biol Med Sci.

[B16] Angen Ø (1997). Taxonomy of the ruminant, porcine and leprine [*Pasteurella*] *haemolytica*-complex. PhD-thesis.

[B17] Christensen H, Bisgaard M, Bojesen AM, Mutters R, Olsen JE (2003). Genetic relationships among avian isolates classified as *Pasteurella haemolytica*, '*Actinobacillus salpingitidis*' or *Pasteurella anatis *with proposal of *Gallibacterium anatis *gen. nov., comb. nov. and description of additional genomospecies within *Gallibacterium *gen. nov. Intl J Syst Evol Microbiol.

[B18] Vogel BF, Jorgensen K, Christensen H, Olsen JE, Gram L (1997). Differentiation of *Shewanella putrefaciens *and *Shewanella alga *on the basis of whole-cell protein profiles, ribotyping, phenotypic characterization, and 16S rRNA gene sequence analysis. Appl Environ Microbiol.

[B19] Dewhirst FE, Paster BJ, Bright PL (1989). *Chromobacterium, Eikenella, Kingella, Neisseria, Simonsiella*, and *Vitreoscilla *species comprise a major branch of the beta group *Proteobacteria *by 16S ribosomal ribonucleic acid sequence comparison: transfer of *Eikenella *and *Simonsiella *to the family *Neisseriaceae*. Int J Syst Bacteriol.

[B20] Paster BJ, Dewhirst FE (1988). Phylogeny of campylobacters, wolinellas, *Bacteroides gracilis*, and *Bacteroides ureolyticus *by 16S ribosomal ribonucleic acid sequencing. Int J Syst Bacteriol.

[B21] Altschul S, Madden T, Schaffer A, Zhang J, Zhang Z, Miller WG, Lipman D (1997). Gapped BLAST and PSI-BLAST: a new generation of protein data-base search programs. Nucleic Acids Res.

[B22] Biberstein EL, Thompson DA (1966). Epidemiological studies on *Pasteurella haemolytica *in sheep. J Comp Path.

[B23] Larsen J, Pedersen AG, Christensen H, Bisgaard M, Angen Ø, Ahrens P, Olsen JE Evolution of the leukotoxin operon in genus *Mannheimia*. J Mol Evol.

[B24] Savelkoul PHM, Aarts HJM, Haas J, Dijkshoorn L, Duim B, Otsen M, Rademaker JLW, Schouls L, Lenstra JA (1999). Amplified fragment length polymorphism analysis: the state of an art. J Clin Microbiol.

[B25] Janssen P, Maquelin K, Coopman R, Tjernberg I, Bouvet P, Kersters K, Dijkshoorn L (1997). Discrimination of *Acinetobacter *genomic species by AFLP fingerprinting. Int J Syst Bacteriol.

[B26] Bojesen AM, Torpdahl M, Christensen H, Olsen JE, Bisgaard M (2003). Genetic diversity of *Gallibacterium anatis *isolates from different chicken flocks. J Clin Microbiol.

